# FLII Modulates the Myogenic Differentiation of Progenitor Cells via Actin Remodeling-Mediated YAP1 Regulation

**DOI:** 10.3390/ijms241814335

**Published:** 2023-09-20

**Authors:** Mai Thi Nguyen, Quoc Kiet Ly, Hyun-Jung Kim, Wan Lee

**Affiliations:** 1 Department of Biochemistry, Dongguk University College of Medicine, 123 Dongdae-ro, Gyeongju 38066, Republic of Korea; nguyenmainhp@gmail.com (M.T.N.); kietly1501@gmail.com (Q.K.L.); hunjung612@naver.com (H.-J.K.); 2 Channelopathy Research Center, Dongguk University College of Medicine, 32 Dongguk-ro, Goyang 10326, Republic of Korea

**Keywords:** FLII, differentiation, myogenesis, mechanotransduction, actin remodeling

## Abstract

The dynamic rearrangement of the actin cytoskeleton plays an essential role in myogenesis, which is regulated by diverse mechanisms, such as mechanotransduction, modulation of the Hippo signaling pathway, control of cell proliferation, and the influence of morphological changes. Despite the recognized importance of actin-binding protein Flightless-1 (FLII) during actin remodeling, the role played by FLII in the differentiation of myogenic progenitor cells has not been explored. Here, we investigated the roles of FLII in the proliferation and differentiation of myoblasts. FLII was found to be enriched in C2C12 myoblasts, and its expression was stable during the early stages of differentiation but down-regulated in fully differentiated myotubes. Knockdown of FLII in C2C12 myoblasts resulted in filamentous actin (F-actin) accumulation and inhibited Yes-associated protein 1 (YAP1) phosphorylation, which triggers its nuclear translocation from the cytoplasm. Consequently, the expressions of YAP1 target genes, including PCNA, CCNB1, and CCND1, were induced, and the cell cycle and proliferation of myoblasts were promoted. Moreover, FLII knockdown significantly inhibited the expression of myogenic regulatory factors, i.e., MyoD and MyoG, thereby impairing myoblast differentiation, fusion, and myotube formation. Thus, our findings demonstrate that FLII is crucial for the differentiation of myoblasts via modulation of the F-actin/YAP1 axis and suggest that FLII is a putative novel therapeutic target for muscle wasting.

## 1. Introduction

Skeletal muscle constitutes approximately 50% of the body mass and subserves various functions such as locomotion, respiration, temperature regulation, and metabolism [1]. Since skeletal myogenesis is critical for muscle development, growth, and regeneration [2], any impairment of this process can lead to muscle atrophy, characterized by loss of muscle quantity, quality, and regenerative capacity [3]. Differentiation of progenitor cells during skeletal myogenesis involves multiple well-ordered steps, viz., withdrawal from the cell cycle, initiating the myogenic transcriptional programs, and undergoing morphological changes, such as elongation, alignment, and fusion, to form myofibrils [2]. Although various signaling ligands and their receptors influence the formation and growth of myofibers, recent evidence indicates a close relationship exists between actin cytoskeletal dynamics and skeletal myogenesis by linking mechanotransduction, Hippo signaling, proliferation, and differentiation [4,5].

Actin is the most abundant intracellular protein and is indispensable for mechanical support, shape alterations, and motility of the cells due to its polymerizing and depolymerizing abilities [6,7,8]. The morphological changes exhibited in myoblasts during differentiation indicate that actin cytoskeleton remodeling is a critical constituent to forming myofibers [2]. Furthermore, this actin remodeling facilitates the activation of transcriptional programs of muscle-specific regulatory factors in differentiation [4,5]. Hence, disrupting the actin remodeling by chemical or other means inhibits myoblast differentiation and myotube formation [9,10,11]. Interestingly, it was recently revealed that actin dynamics play significant roles in the regulation of the cell cycle and proliferation, which is intimately linked to myoblast differentiation [7]. Mechanistically, the remodeling of filamentous actin (F-actin) reciprocally regulates the Hippo signaling pathway, which modulates tissue and organ size via Yes-associated protein 1 (YAP1) [4,5]. YAP1 functions as a transcriptional coactivator of the TEAD transcription factor and influences various cellular processes, including proliferation, migration, differentiation, and survival [12]. Therefore, the accumulation of F-actin induces mechanotransduction-derived YAP1 activation in the Hippo signaling pathway and enhances cell cycle progression and proliferation [13]. In this respect, it is interesting to note that several proteins involved in actin remodeling between globular actin (G-actin) and F-actin have been suggested as critical regulators in myogenesis [14,15,16].

Actin remodeling protein Flightless I (FLII) is a member of the gelsolin family of actin-binding proteins (ABPs) and a nuclear receptor coactivator that links signal transduction with cytoskeletal dynamics [17]. FLII is highly expressed in *Drosophila*, mice, and human skeletal and cardiac muscle and is a multifunctional protein that regulates actin remodeling [18]. FLII colocalizes with the regulatory proteins involved in cytoskeletal dynamics, including Rho, Ras, and Cdc42 [18,19], directly binds to actin via its gelsolin domain [18], and suppresses actin polymerization [20]. Therefore, FLII overexpression reduces F-actin formation in fibroblasts [20], whereas silencing FLII expression in *Drosophila* indirect flight muscles results in disorganized myofibrils containing high F-actin levels [21,22]. However, although FLII is known to modulate actin filament assembly, the functional significance of FLII during the differentiation of myogenic progenitor cells remains to be determined.

Our current study aimed to examine the role of FLII in the proliferation and differentiation of myogenic progenitor cells. We assessed FLII expression in various tissues and analyzed changes in its expression during the myogenic differentiation of C2C12 myoblasts. Additionally, we investigated the impact of FLII knockdown on F-actin formation, myogenic factors expressions, differentiation, and myotube formation. Moreover, we explored the underlying mechanisms responsible for the impaired myogenic differentiation resulting from FLII depletion by analyzing YAP1 activation, cell cycle progression, and cell proliferation. This study reveals that FLII plays a critical role in myogenic differentiation and myotube formation through the modulation of the F-actin/YAP1 axis.

## 2. Results

### 2.1. FLII Expression Is Modulated during Myoblast Differentiation

**Figure 1:**
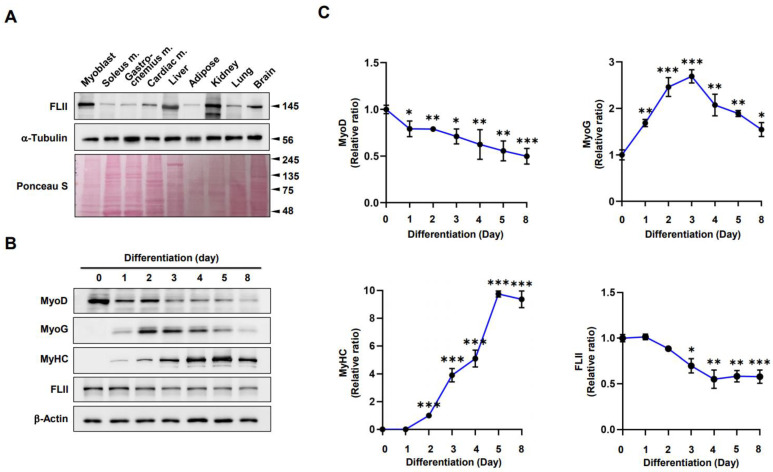
FLII expressional patterns in mouse tissues and during C2C12 differentiation. (**A**) Relative expression levels of FLII in C2C12 myoblasts and various tissues of C57BL/6 mice were analyzed via immunoblotting using α-Tubulin as the loading control. (**B**) C2C12 myoblasts were differentiated for 8 days, and the expressions of MyHC, MyoD, MyoG, and FLII were assessed via immunoblotting. (**C**) Protein levels in (**B**) were quantified via densitometry and normalized versus β-actin. Results are presented as relative ratios where degrees of differentiation on day 0 (MyoD and FLII), day 1 (MyoG), or day 2 (MyHC) were set to one. Results are presented as means ± SEMs (*n* > 3), and statistical significances are represented by *, *p* < 0.05; **, *p* < 0.01; ***, *p* < 0.001 vs. differentiation day 0.

Actin dynamics and the regulation of F-actin by ABPs like FLII are critical for maintaining the structure and function of muscle [18]. Prior to investigating the role of FLII on myogenic differentiation, we determined the levels of FLII in the mouse tissues, including C2C12 myoblasts; skeletal muscles; and liver (Figure 1A). FLII was expressed in most tissues, including muscle, liver, brain, and lung, and interestingly, FLII protein levels were higher in myoblasts but lower in fully differentiated muscle tissues, such as soleus, gastrocnemius, and cardiac muscles, than in other tissues. We also examined the changes in FLII expression during myoblast differentiation. C2C12 cells were differentiated for up to 8 days, and protein levels of FLII, myogenic regulatory transcription factors (i.e., MyoD and MyoG), and differentiation marker myosin heavy chain (MyHC) were determined by immunoblotting (Figure 1B,C). Under the experimental conditions used, the expression levels of MyoD (the earliest myogenic commitment marker) gradually declined after the onset of differentiation. In contrast, the expression levels of MyoG (a marker of differentiation initiation) were significantly upregulated, peaked on differentiation day 3, and then markedly decreased. The pattern of FLII expression was somehow similar to that of MyoD (Figure 1B,C). During the early stages of differentiation (D0–D2), FLII expression remained stable but progressively declined during myoblasts to myotubes differentiation until differentiation day 8 (D8). Since FLII and MyoG expression patterns were inversely related, and FLII is involved in actin cytoskeletal dynamics, we postulated that FLII might be required during the early differentiation stage.

### 2.2. Knockdown of FLII Augments F-Actin and Nuclear YAP1 Levels in Myoblasts

**Figure 2:**
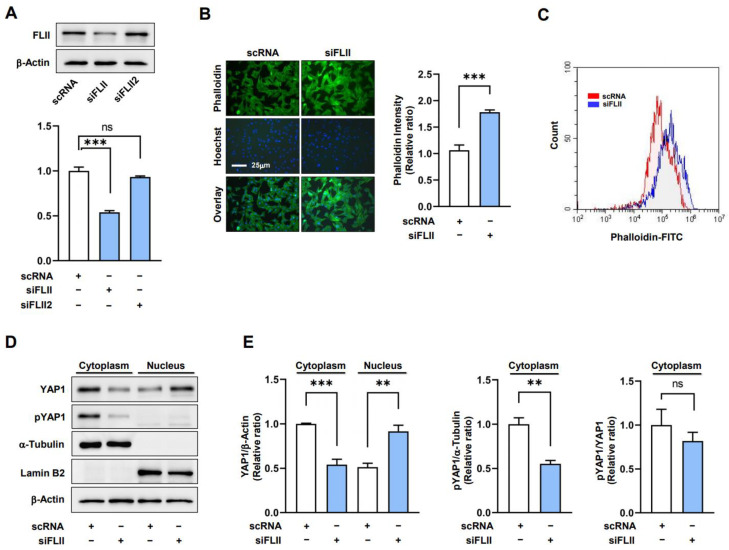
FLII knockdown induced the accumulation of F-actin and nuclear YAP1. C2C12 cells were transfected with 200 nM FLII siRNA (siFLII or siFLII2) or control (scRNA) for 24 h. (**A**) FLII protein levels were determined via immunoblotting. (**B**) Immunostaining was performed using F-actin (FITC-Phalloidin, green) and Hoechst 33342 (blue, a nuclear stain). Scale bar: 25 μm. Phalloidin intensities were quantified using ImageJ software 1.54f. (**C**) Determination of F-actin contents in FITC-phalloidin stained cells via flow cytometry. (**D**,**E**) YAP1 and phosphor-YAP1 (pYAP1) immunoblots were analyzed in the cytoplasmic and nuclear fractions. α-Tubulin and Lamin B were used as cytoplasmic and nuclear fraction markers, respectively. Results are relative ratios versus Lamin B or α-Tubulin and are presented as means ± SEMs (*n* > 3). Statistical significances are represented by **, *p* < 0.01 or ***, *p* < 0.001 vs. scRNA controls. ns: no significance.

Previous studies have reported that FLII knockdown increased F-actin formation and disrupted myofibril organization [21] and that F-actin accumulation is linked to cell cycle progression and proliferation via the nuclear translocation of YAP1 [13]. Hence, we hypothesized that FLII knockdown might induce the nuclear translocation of YAP1 by enhancing F-actin accumulation in C2C12 myoblasts. Initially, we transfected myoblasts with scRNA control or two siRNAs against FLII (siFLII or siFLII2) and allowed cells to grow for 24 h in GM. Transfection with siFLII reduced the protein level of FLII by ~50% compared to scRNA (Figure 2A). Therefore, we used siFLII to deplete the FLII protein expression. Under this experimental condition, we investigated whether FLII knockdown increases F-actin in myoblasts. Phalloidin staining in fluorocytometry showed that siFLII markedly increased F-actin levels by ~60% versus scRNA (Figure 2B). Moreover, increased F-actin formation by siFLII was also confirmed via FACS analysis (Figure 2C). Immunoblot analysis showed total actin levels were unaffected by treatments, which indicated siFLII-induced increases in F-actin were caused by insufficient actin depolymerization resulting from FLII suppression. We also found that transfection of siFLII led to a dramatic enhancement in the contraction of C2C12 cells, as determined by the reduction in gel surface area (Supplementary Figure S1). Furthermore, Y-27632 (a Rho-associated protein kinase (ROCK) inhibitor) effectively suppressed the siFLII-induced contraction of C2C12 cells. Thus, it is suggested that the contractility triggered by FLII knockdown primarily depends on ROCK activity.

F-actin has been known to facilitate YAP1 nuclear translocation, which activates the gene expression involved in cell proliferation by inhibiting YAP1 phosphorylation [23]. Hence, we next investigated whether FLII knockdown increases the nuclear localization of YAP1 in C2C12 cells by determining nuclear YAP1 levels and the extent of YAP1 phosphorylation at Ser127. As was expected, C2C12 cells transfected with siFLII exhibited markedly increased nuclear YAP1 levels concomitant with the decreased YAP1 phosphorylation in the cytoplasm (Figure 2D,E). Thus, these results suggest that FLII knockdown inhibits YAP1 phosphorylation and consequently facilitates the nuclear translocation of YAP1 from the cytoplasm. The YAP1 translocation observed in the siFLII-transfected cells was restored to levels comparable to those seen in the scRNA controls when treated with Y-27632 (Supplement Figure S1B), indicating that ROCK provides critical mediating mechanisms for FLII knockdown-induced YAP1 nuclear translocation in myoblasts.

### 2.3. FLII Knockdown Enhances Myoblast Proliferation

**Figure 3:**
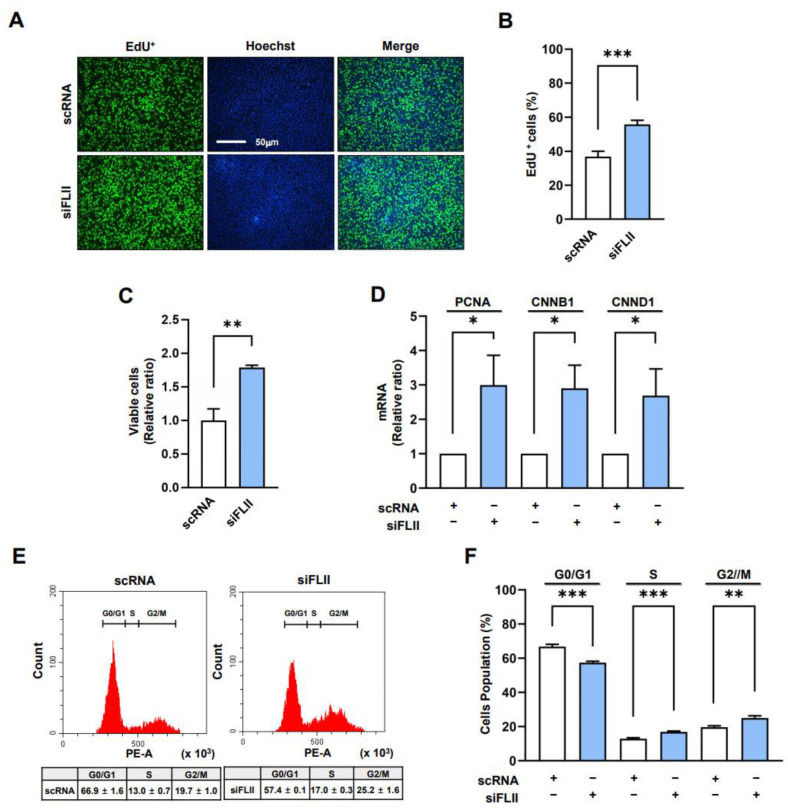
FLII knockdown facilitated myoblast proliferation and cell cycle progression. C2C12 cells were transfected with FLII siRNA (siFLII) or control (scRNA) for 24 h. (**A**) Cells undergoing DNA replication were immunostained with EdU (green) and Hoechst 33342 (blue). Scale bar: 50 µm. (**B**) Proportions of EdU-positive cells were analyzed using ImageJ software. (**C**) Viable cells were quantitated using a cell viability assay kit. (**D**) Relative mRNA expressions of PCNA, CCNB1, and CCND1 were determined via *q*RT-PCR and normalized versus U6. (**E**,**F**) Cell cycle assay determined percentages of cells in the G0/G1, S, and G2/M phases. *q*RT-PCR and immunoblot results are normalized versus scRNA controls. Results are presented as means ± SEMs (*n* > 3). Statistical significances are represented by *, *p* < 0.05; **, *p* < 0.01; ***, *p* < 0.001 vs. scRNA controls. ns: no significance.

Given that the nuclear translocation of YAP1 promotes cell cycle progression and proliferation [24,25], we next investigated whether FLII knockdown stimulates cell cycle progression and proliferation in C2C12 myoblasts. After transfecting the myoblasts with siFLII or scRNA control for 24 h, an EdU incorporation assay and viable cell analysis were conducted to measure cell proliferation. FLII knockdown using siFLII substantially elevated the proportion of EdU-positive cells (Figure 3A,B) and viable cells (Figure 3C) by ~1.6-fold and ~1.7-fold, respectively, when compared with scRNA controls, demonstrating that FLII depletion promoted myoblast proliferation. The enhanced cell proliferation by FLII knockdown was rescued to the scRNA control levels by Y-27632 treatment (Supplementary Figure S1C). In addition, we determined the transcript levels of PCNA, CCNB1, and CCND1, which are known to induce cell cycle progression and proliferation [26], to verify the effect of siFLII on cell proliferation. In line with our results from the EdU incorporation and viable cell analysis, the mRNA of PCNA, CCNB1, and CCND1 were dramatically increased by FLII knockdown versus scRNA controls (Figure 3D). Next, we analyzed the cell cycle to determine whether depletion of FLII promotes cell cycle progression. Flow cytometry analysis revealed that siFLII transfections elevated the proportions of cells in the S and G2/M phases and decreased the proportion of cells in the G0/G1 phase (Figure 3E,F), indicating that FLII knockdown facilitates cell cycle progression. Collectively, these observations indicate that FLII depletion facilitates cell cycle progression and myoblast proliferation.

### 2.4. FLII Is Required for Myogenic Factors Expressions and Myoblast Differentiation

**Figure 4:**
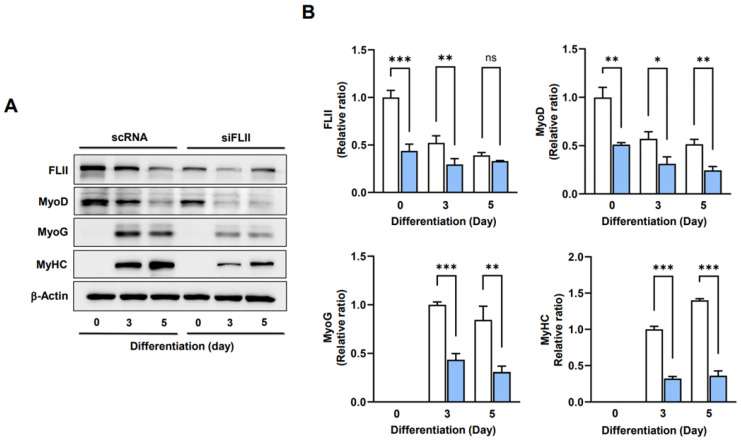
FLII knockdown inhibited the expressions of myogenic regulatory factors. C2C12 cells were transfected with FLII siRNA (siFLII, blue column) or control (scRNA, white column) and cultured in a differentiation medium for three or five days. (**A**) Representative immunoblots of FLII, MyoD, and MyoG proteins. (**B**) Quantification of protein levels in (**A**) normalized versus β-actin. Results are presented as means ± SEMs (*n* > 3). Statistical significances are represented by *, *p* < 0.05; **, *p* < 0.01; ***, *p* < 0.001 vs. scRNA controls. ns: no significance.

As the depletion of FLII enhanced cell cycle progression, an unfavorable condition for the differentiation of progenitor cells [2], we considered that FLII knockdown might suppress myogenic factors expressions and thereby hinder myoblast differentiation. The protein levels of MyoD and MyoG in C2C12 cells were determined on differentiation days 0, 3, and 5 after transfection with scRNA or siFLII. siFLII transfection resulted in a significant reduction in the protein levels of FLII, MyoD, and MyoG (Figure 4A,B), and as a result, levels of MyHC were markedly lower in siFLII-transfected cells than in scRNA controls (Figure 4A,B). These results suggest that FLII is required to initiate the expressions of myogenic transcription factors.

**Figure 5:**
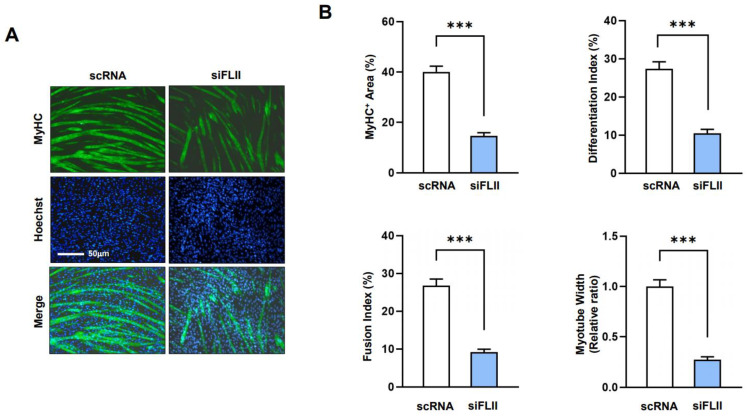
FLII knockdown suppressed myogenic differentiation and myotube formation. C2C12 cells were transfected with 200 nM FLII siRNA (siFLII) or control (scRNA) and differentiated for up to five days. (**A**) Cells were subjected to fluorescence analysis using MyHC antibody (MF20, green) and Hoechst 33342 (blue). Scale bar: 50 μm. (**B**) MyHC-positive areas, differentiation indices, fusion indices, and myotube widths were determined as described in Section 4. Results are presented as means ± SEMs (*n* > 3). Statistical significances are represented by ***, *p* < 0.001 vs. scRNA controls.

Next, myoblasts transfected with scRNA or siFLII were allowed to differentiate for five days in DM, and differentiation and myotube formation were assessed via immunocytochemistry using MyHC antibody and image analysis. FLII knockdown was found to impede differentiation and myotube formation dramatically, as determined using immunocytochemistry, areas of MyHC-positive cells, indices for differentiation and fusion, and myotube widths (Figure 5A,B). Therefore, these results suggest that FLII is essential for the myogenic differentiation and myotube formation of progenitor cells.

## 3. Discussion

Accumulating evidence suggests that FLII is an essential regulator of actin cytoskeletal dynamics [20,21,22], which has a multifaceted association with the regulation of myogenesis [4,5,6,7]. However, the biological role played by FLII during the differentiation of progenitor cells has not been explored. This study reveals the following roles of FLII in myoblasts: (i) FLII expression is modulated during the myogenic differentiation of C2C12 myoblasts, (ii) knockdown of FLII expression results in the accumulation of F-actin and increases nuclear YAP1 levels in myoblasts, (iii) FLII depletion promotes cell cycle progression and proliferation, and (iv) FLII knockdown suppresses the expression of myogenic transcription factors and impairs myoblast differentiation. Thus, our findings suggest FLII regulates myogenic differentiation through the F-actin/YAP1 axis.

During the formation of myofibers from progenitor cells, morphological changes in the cell shape and diverse signaling cascades for regulating the cell cycle and myogenic gene expression are required [2]. In this process, actin cytoskeletal dynamics provide the physical changes required for membrane reconfiguration and trigger signal transductions that induce the transcriptional activation of myogenic genes [2]. Hence, actin remodeling and its regulatory proteins play essential roles in myogenesis [4,5]. From this point of view, it is interesting that FLII, an actin remodeling protein inhibiting actin polymerization without possessing actin-severing activity [20,21,22], is indispensable for myogenic differentiation. FLII was expressed ubiquitously in various tissues, including skeletal and cardiac muscle, and abundantly expressed in C2C12 myoblasts (Figure 1). This observation concurs with those of a previous study, in which FLII protein was found to be abundant in *Drosophila*, mouse, and human skeletal and cardiac muscle [18]. Since FLII is known to be necessary for embryogenesis and myogenesis in Drosophila [18,19,27], we monitored its expression during the differentiation of C2C12 myoblasts (Figure 1). The expression of FLII was stable for the early stage of differentiation (D0-D2) and subsequently down-regulated gradually as C2C12 myoblasts differentiated into myotubes. Moreover, knockdown of FLII by siRNA drastically impaired the expressions of myogenic genes and the differentiation of myoblasts. These findings caused us to hypothesize that FLII plays an essential role in myogenic differentiation in progenitor cells.

Consistent with this hypothesis, the knockdown of FLII expression facilitated cell cycle progression and myoblast proliferation, ultimately leading to impaired myogenic differentiation. The inverse relationship between myoblast proliferation and differentiation into myotubes is a well-known phenomenon in myogenesis [2,28]. Although the proliferation of progenitor cells might be expected to promote myogenesis, it actually impedes the differentiation process because myoblast differentiation and subsequent myotube formation require the activations of specific myogenic regulatory factors, which require cell cycle exit [2,28]. Thus, the promotion of myoblast proliferation delays the timing of cell cycle exit and consequently prevents myoblasts from differentiating into muscle cells. From this perspective, it is worth noting that several recent studies have reported the inverse relationship between FLII expression and cell proliferation in various cells. For example, FLII knockdown by shRNA increased the cell migration and proliferation of NIH/3T3 fibroblasts [20], and FLII-deficient +/− mice exhibited enhanced epithelial cell proliferation, migration, and improved wound healing [29]. Conversely, cell proliferation and epithelial migration were reduced in FLII-overexpressing mice, leading to significantly impaired wound healing [29,30]. Also, FLII plays a tumor-suppressive role and is considered a critical determinant of prostate cancer resistance to endocrine-based therapies [31]. Thus, it is suggested that FII depletion during the early stage of differentiation increases cell proliferation, impedes cell cycle withdrawal, and, thereby, impairs the myogenic differentiation of C2C12 cells.

**Figure 6:**
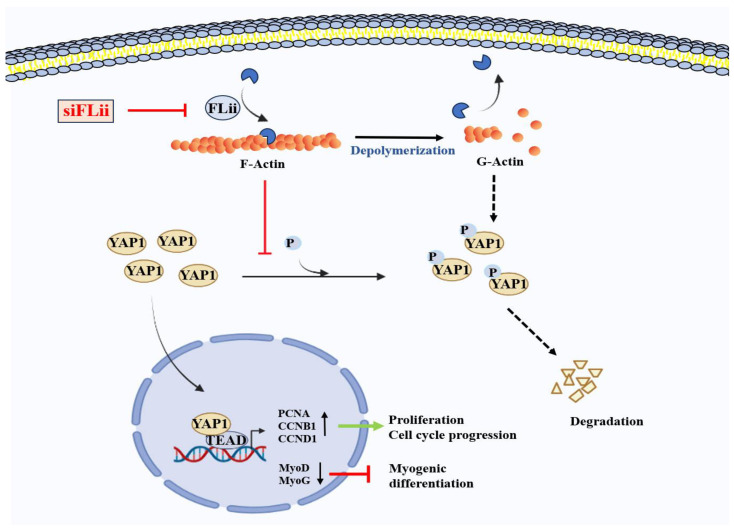
Role of FLII in the regulation of actin remodeling, YAP1 localization, cell proliferation, and myogenic differentiation.

Then what is the underlying molecular mechanism responsible for myoblast proliferation and impaired differentiation by the depletion of FLII? In progenitor cells, cytoskeletal changes involving actin assembly, disassembly, and rearrangement play integral roles in the cell cycle, cell proliferation, and the activations of myogenic transcription factors [6,32]. Furthermore, actin remodeling critically regulates YAP1 in the Hippo signaling pathway through mechanotransduction [33]. YAP1 is a transcriptional coactivator that regulates the expression of target genes required for cell proliferation, tissue growth, and organ development [12]. MST1/2 and LATS1/2 kinases in the Hippo signaling pathway promote the phosphorylation of YAP1, which leads to proteasomal degradation of YAP1 and subsequent inhibition of YAP1 nuclear translocation [34]. It has been suggested that F-actin stress fiber can promote the nuclear translocation of YAP1 by regulating MST1/2 activity. F-actin-bound MST1/2 is inactive, while the decline of F-actin results in MST1/2 activation, which then phosphorylates LATS1/2 kinases, leading to the phosphorylation and degradation of YAP1 [35]. Furthermore, the recent discovery of Angiomotin (Amot) clarified the relationship between F-actin and the Hippo pathway. Amot contains a conserved binding domain capable of interacting with F-actin and YAP1 [36]. Interestingly, LATS-mediated Amot phosphorylation prevented Amot binding to F-actin but promoted its association with YAP1, which led to the retention of YAP1 in the cytoplasm and its subsequent degradation by proteasomes [37]. Thus, F-actin accumulation inhibits Hippo signaling and facilitates the nuclear translocation and activation of YAP1 as a consequence of the mechanotransduction mechanism [23]. In this regard, the knockdown of F-actin-capping/severing proteins, such as Gelsolin, Capz, and Cofilin, also led to the mechanotransduction-induced nuclear translocation of YAP1 and provoked cell proliferation [13,38]. In conclusion, as illustrated in Figure 6, we have revealed that siFLII dramatically increased F-actin levels by suppressing FLII expression, reducing YAP1 phosphorylation and facilitating the nuclear translocation of YAP1, which stimulates the expressions of YAP1 target genes involved in cell cycle progression and proliferation. Therefore, our study suggests that FLII plays a crucial role in myoblast proliferation and differentiation through the regulation of the F-actin/YAP1 axis in the Hippo signaling pathway.

## 4. Materials and Methods

### 4.1. Cell Culture and Differentiation

C2C12 cells (murine myogenic progenitor cells) were obtained from the ATCC (Manassas, VA, USA) and cultured in growth medium (GM) [high glucose (25 mM) DMEM containing FBS (10%) and penicillin/streptomycin (1%) (Gibco, Carlsbad, CA, USA)] at 3 °C in a 5% CO_2_ atmosphere. When cells were 80% to 90% confluent (day 0), GM was replaced with differentiation medium (DM) [high glucose (25 mM) DMEM containing horse serum (2%, Gibco) and penicillin/streptomycin (1%)]. DM was refreshed every 24 h.

### 4.2. Oligonucleotide Transfection

C2C12 cells were seeded on 35 mm dishes at ~1.3 × 10^5^ cells/dish. The next day, cells were transiently transfected with scrambled control RNA (scRNA) or FLII siRNA (siFLII) (Genolution, Seoul, Republic of Korea) using Lipofectamine 2000 (Invitrogen, Thermofisher Scientific, Waltham, MA, USA), incubated in DMEM for 4 h, and then maintained in GM for 24 h, according to the manufacturer’s instructions. The sequences of oligonucleotides are shown in Table S1.

### 4.3. Real-Time Quantitative Reverse Transcription PCR (qRT-PCR)

Cells were lysed using Qiazol reagent (Qiagen, Hilden, Germany), and total RNA was isolated using the miRNeasy Mini Kit (Qiagen). cDNA was synthesized from 1µg RNA using the miScript II RT Kit (Qiagen). Relative mRNA expressions were determined via *q*RT-PCR using SYBR Green I (Promega, Madison, WI, USA) in a LightCycler 480 (Roche Applied Science, Basel, Switzerland) and normalized versus GAPDH using the 2^−ΔΔCt^ method. The primer sequences and reaction conditions for *q*RT-PCR are shown in Table S2.

### 4.4. Cytoplasmic and Nuclear Extraction

After reaching 80–90% confluence in 35 mm dishes, C2C12 cells were harvested using trypsin/EDTA (Gibco) and centrifuged at 3000 rpm for 5 min at 4 °C. Pellets were rinsed with pre-cold PBS and extracted using NE-PER™ Nuclear and Cytoplasmic Extraction Reagents (Thermo Fisher Scientific, Waltham, MA, USA) to separate cytoplasmic and nuclear fractions. Briefly, pellets were resuspended in 100 µL CER I and incubated on ice for 30 min; 5.5 µL CER II was added for the last minute of this incubation. Samples were centrifuged at 13,000 rpm for 15 min at 4 °C, and supernatants (cytoplasmic fractions) were collected. Pellets were resuspended in 50 µL of ice-cold NER solution, incubated for 40 min, centrifuged, and supernatants (nuclear fractions) were collected. All steps were done on ice, and cytoplasmic and nuclear fractions were immunoblotted, as described below.

### 4.5. Immunoblot Analysis 

Cells were lysed with a lysis buffer (PBS buffer supplemented with 0.2 mM PMSF, 2% Triton-X, and 1% phosphatase inhibitor cocktail II (Sigma-Aldrich, St. Louis, MO, USA)). Protein concentrations were determined using the Bradford assay, and equal amounts of protein (20 µg/well) were resolved via SDS-PAGE and transferred to nitrocellulose membranes. After blocking with 5% skim milk in TTBS solution (0.5% TBS-Tween 20) for 1 h, membranes were incubated overnight at 4 °C with primary antibodies (Table S3). The next day, membranes were washed with TTBS six times and treated with a secondary antibody (1: 10,000 dilution) at room temperature (RT) for 1 h. Proteins were visualized using a chemiluminescent Femto reagent (Thermo Fisher Scientific) and a Fusion Solo S imaging system (Vilber Lourmat, Paris, France). Western blots were quantified using Evolution-Capt v18.10 software supplied by Vilber Lourmat (Paris, France).

### 4.6. Immunofluorescence Analysis

Differentiated C2C12 cells were fixed in 4% paraformaldehyde for 10 min, permeabilized with 0.3% Triton X-100 for 15 min, and blocked in 3% bovine serum albumin in PBS for 2 h at RT. Cells were then immunostained with anti-myosin heavy chain (MyHC) antibody (1:100 dilution) overnight at 4 °C, washed three times with PBS, and incubated with a secondary antibody (Alexa 488, Invitrogen) for 1 h at RT. For F-actin staining, fixed cells were incubated with FITC-coupled phalloidin (P5282, Sigma, St. Louis, MO, USA) for 20 min at RT. Nuclei were visualized using Hoechst 33342 (Invitrogen). Fluorescent images of five randomly chosen microscopic fields of the experiments performed in triplicate were captured using a Leica fluorescence microscope (Microsystems, Mannheim, Germany). To assess degrees of differentiation, ImageJ software was used to determine the frequencies and total counts of nuclei present in MyHC-positive cells. Degrees of differentiation were assessed using differentiation (number of nuclei in MyHC^+^ cells/total nuclei) and fusion indices (number of myotubes with ≥3 nuclei/total nuclei).

### 4.7. Cell Proliferation Assay 

C2C12 cells were seeded onto 8-well chamber slides at 10^4^ cells/well, and cell proliferation ability was determined using the Click-iT™ EdU Cell Proliferation Kit (Invitrogen) after 24 h of transfection. In brief, transfected cells were labeled with EdU-incorporation solution (10 µM) in GM for 4 h in a 37 °C incubator, then fixed and permeabilized as described in Section 4.6. Cells were then washed with PBS, treated with 300 µL of Click-iT^®^ reaction cocktail, and counterstained for 15 min with Hoechst 33342. Images were captured using a Leica fluorescent microscope. EdU-positive cell percentages of total cells were calculated using at least five randomly selected images of three independent experiments.

### 4.8. Cell Viability

C2C12 cells were grown in 96-well plates at 10^3^ cells/well, transfected with indicated oligonucleotides using Lipofectamine 2000 (Invitrogen) for 24 h, and cultured in GM containing 10 μL of Quanti-max™ WST-8 Cell viability Assay Kit solution (BioMax, Seoul, Republic of Korea) for 4 h at 37 °C. A microplate reader (Model 680, Bio-Rad, Hercules, CA, USA) was used to measure cell viability at 450 nm.

### 4.9. Flow Cytometry

Transfected cells were harvested into 1.5 mL tubes using trypsin/EDTA (Gibco) and centrifuged at 3000 rpm for 5 min at 4 °C. Pellets were fixed overnight in 70% ethanol at 4 °C, washed with PBS, and cells were stained using 0.5 mL of Cell Cycle kit solution (C03551, Beckman Coulter, Pasadena, CA, USA) for the cell cycle assay or 30 µM of FITC-coupled phalloidin solution (P5282, Sigma) for the F-actin analysis. Cell cycle assays and F-actin measurements were conducted after incubating cells for 20 min in the dark using a CytoFLEX unit (Beckman Coulter, USA).

### 4.10. Statistical Analysis

Results are presented as the means ± SEM of at least three independent experiments. The analysis was conducted using the one-way independent Student’s *t*-test for unpaired data.

## Data Availability

The data presented in this study are available on request from the corresponding author.

## Supplementary Materials

The following supporting information can be downloaded at https://www.mdpi.com/article/10.3390/ijms241814335/s1.
