# Supplementary material for: FLII Modulates the Myogenic Differentiation of Progenitor Cells via Actin Remodeling-Mediated YAP1 Regulation
Source: Int J Mol Sci. 2023 Sep 20;24(18):14335. doi: 10.3390/ijms241814335 (PMC10531566; doi:10.3390/ijms241814335)
Supplement: Supplementary file 1 [file ijms-24-14335-s001.zip › ijms-2534632-supplementary/ijms-2534632-supplementary figure.pdf]

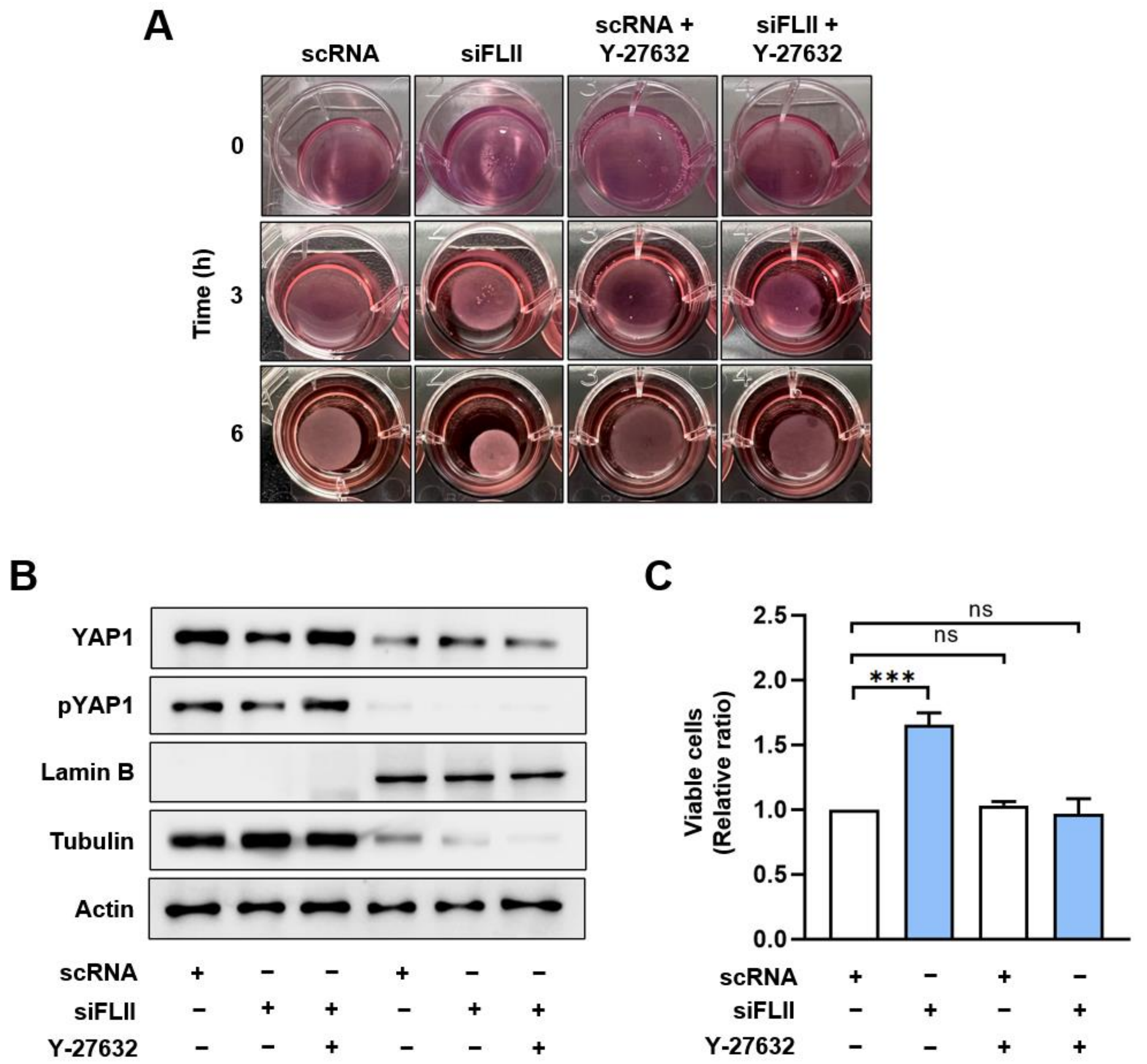

Supplementary Figure 1. ROCK inhibitor Y-27632 inhibits FLII knockdown-induced contraction, YAP1 nuclear translocation, and proliferation in myoblasts. C2C12 cells were transfected with 200 nM FLII siRNA (siFLII) or control (scRNA) for 24 h in the presence or absence of Y-27632 (10μM). **(A)** Collagen gel contraction assay. **(B)** YAP1 and phosphor-YAP1 (pYAP1) immunoblots were analyzed in the cytoplasmic and nuclear fractions. α-Tubulin and Lamin B were used as cytoplasmic and nuclear fraction markers, respectively. **(C)** Viable cells were quantitated using a cell viability assay kit. Statistical significances are represented by ***, $P<0.001$ vs. scRNA controls. ns: no significance.

Results

To analyze the effect of FLII knockdown on cell contraction, C2C12 myoblasts transfected with scRNA

or siFLII for 24 h were plated to adhere to collagen gels in the presence or absence of Y-27632 (the ROCK inhibitors, 10μM). As shown in Supplement Figure 1A, transfection of siFLII led to a dramatic enhancement in the contraction of C2C12 cells, as determined by the reduction in gel surface area. Therefore, it is suggested that FLII knockdown stimulates the contraction of myoblasts. Furthermore, Y-27632 effectively suppressed the siFLII-induced contraction of C2C12 cells. Thus, it is evident that the contraction triggered by FLII knockdown primarily depends on ROCK activity.

In this experimental context, we also examined the effect of Y-27632 on two other aspects: YAP1 nuclear translocation and cell proliferation induced by FLII knockdown. Supplement Figure 1B an 1C shows that YAP1 nuclear translocation and cell proliferation in the siFLII-transfected cells were restored to levels comparable to those seen in the scRNA controls when treated with Y-27632. Hence, it is demonstrated that ROCK provides critical mediating mechanisms for FLII knockdown-induced contraction, YAP1 nuclear translocation, and proliferation in myoblasts.

Methods and Materials for Supplement Figure 1

Collagen gel contraction assay

C2C12 cells were transfected with 200nM siFLII or scRNA in the presence or absence of Y-27632 (20 μM, Cayman Chemical, Michigan, USA) for 24 h. The next day, cells were harvested using Trypsin/EDTA(Gibco), centrifuge at 4°C, 3000 rpm, 5 min, and the cell concentration was adjusted to 125x10^{5} cells/ml. Then, 400 μl of cell suspension and 200 μl of 3mg/ml Collagen I solution (Gibco) were mixed thoroughly in an e-tube. Following neutralization using NaOH 1M with appropriate volume, 600 μl of cell suspension and collagen mixture were gently transferred to each well of a 24-well plate and allowed to solidify at room temperature for 20 min. After polymerization, gels were dissociated from its mold by gently running the 200 μl pipet tip along the gel edges. Finally, gels were added 500 μl GM and transferred in a 37°C incubator. The gel images were taken at 0, 3, and 6 h, and the gel surface areas were subsequently calculated from scanner-generated digital images using ImageJ.

Detailed methods for cytoplasmic and nuclear extraction, immunoblotting, and cell proliferation assays were described in the main text.
