# Supplementary material for: FLII Modulates the Myogenic Differentiation of Progenitor Cells via Actin Remodeling-Mediated YAP1 Regulation
Source: Int J Mol Sci. 2023 Sep 20;24(18):14335. doi: 10.3390/ijms241814335 (PMC10531566; doi:10.3390/ijms241814335)
Supplement: Supplementary file 1 [file ijms-24-14335-s001.zip › ijms-2534632-supplementary/ijms-2534632-supplementary tables.pdf]

Table S1. Oligonucleotide sequences for transfection

Gene	Oligonucleotide sequence (5´-3´)
scRNA (control RNA)	UCACAACCUCCUAGAAAGAGUAGA
siFLII	CUGAACUGGGAGAUCUACU
siFLII2	GCCCACGGGAGCUGGAGAAU

Table S2. Primer list and conditions for *q*RT-PCR

Gene	Primer sequence (5´-3´)		Product size	Annealing Temperature	Concentration		Cycle
					cDNA	Primer	
U6	F.P	CTCGCTTCGGCAGCACA	100	58	2 ng/μl	0.5 μM	40
	R.P	AACGCTTCACGAATTTGCGT					
PCNA	F.P	GAACCTGCAGAGCATGGACTC	201	58			
	R.P	GGTGTCTGCATTATCTTCAGCCC					
CCND1	F.P	ACCAATCTCCTCAACGACCG	228	58			
	R.P	ACGGAAGGGAAGAGAAGGG					
CCNB1	F.P	GAGCTATCCTCATTGACTGG	125	58			
	R.P	CATCTTCTTGGGCACACAAC					

Table S3. Antibodies list

Antibody	Type	Targeted species	Manufacturer	Cat. No.	Dilution ratio*
FLII	Monoclonal	Mouse	Santa Cruz Biotechnology, Dallas, TX, USA	sc-21716	1:5,000
MyHC	Monoclonal	Mouse	DSHB, Iowa, IA, USA	MF20	1:1,000
MyoD	Monoclonal	Mouse	Santa Cruz Biotechnology, Dallas, TX, USA	sc-377460	1:1,000
MyoG	Monoclonal	Mouse	Santa Cruz Biotechnology, Dallas, TX, USA	sc-12732	1:1,000
YAP1	Monoclonal	Rabbit	Cell Signaling Technology, Danvers, MA, USA	14074S	1:10,000
pYAP1	Polyclonal	Mouse	Cell Signaling Technology, Danvers, MA, USA	4911S	1:10,000
Lamin B2	Monoclonal	Rabbit	Abcam, Cambridge, United Kingdom	ab151735	1:2,500
α-Tubulin	Monoclonal	Mouse	DSHB, Iowa, IA, USA	12G10	1:2,000
β-actin	Monoclonal	Rabbit	Sigma-Aldrich Chemical, St. Louis, USA	A2066	1:10,000
Antibodies HRP-linked anti-rabbit IgG			Cell Signaling Technology, Danvers, MA, USA	#7074	1:10,000
Goat anti-mouse(H+L)			Thermofisher Sci., Waltham, MA, USA	#32430	1:2,000

*All blots were visualized using a Femto reagent (Thermofisher Scientific).
